# Correlation of Lung Collapse and Gas Exchange - A Computer Tomographic Study in Sheep and Pigs with Atelectasis in Otherwise Normal Lungs

**DOI:** 10.1371/journal.pone.0135272

**Published:** 2015-08-10

**Authors:** Samuel J. Wolf, Alexander P. Reske, Sören Hammermüller, Eduardo L. V. Costa, Peter M. Spieth, Pierre Hepp, Alysson R. Carvalho, Jens Kraßler, Hermann Wrigge, Marcelo B. P. Amato, Andreas W. Reske

**Affiliations:** 1 Department of Anaesthesiology and Intensive Care Medicine, University Hospital Leipzig, Leipzig, Germany; 2 Anaesthesiology and Intensive Care Medicine, Fachkrankenhaus Coswig, Coswig/Meißen, Germany; 3 Cardio-Pulmonary Department, Pulmonary Division, Hospital das Clínicas, University of São Paulo, São Paulo, Brazil; 4 Research and Education Institute, Hospital Sírio Libanês, São Paulo, Brazil; 5 Department of Anaesthesiology and Intensive Care Medicine, University Hospital *Carl Gustav Carus*, Dresden, Germany; 6 Department of Orthopedic, Trauma and Plastic Surgery, University Hospital Leipzig, Leipzig, Germany; 7 Carlos Chagas Biophysics Institute, Federal University of Rio de Janeiro, Rio de Janeiro, Brazil; University of Bari, ITALY

## Abstract

**Background:**

Atelectasis can provoke pulmonary and non-pulmonary complications after general anaesthesia. Unfortunately, there is no instrument to estimate atelectasis and prompt changes of mechanical ventilation during general anaesthesia. Although arterial partial pressure of oxygen (PaO_2_) and intrapulmonary shunt have both been suggested to correlate with atelectasis, studies yielded inconsistent results. Therefore, we investigated these correlations.

**Methods:**

Shunt, PaO_2_ and atelectasis were measured in 11 sheep and 23 pigs with otherwise normal lungs. In pigs, contrasting measurements were available 12 hours after induction of acute respiratory distress syndrome (ARDS). Atelectasis was calculated by computed tomography relative to total lung mass (M_total_). We logarithmically transformed PaO_2_ (lnPaO_2_) to linearize its relationships with shunt and atelectasis. Data are given as median (interquartile range).

**Results:**

M_total_ was 768 (715–884) g in sheep and 543 (503–583) g in pigs. Atelectasis was 26 (16–47) % in sheep and 18 (13–23) % in pigs. PaO_2_ (FiO_2_ = 1.0) was 242 (106–414) mmHg in sheep and 480 (437–514) mmHg in pigs. Shunt was 39 (29–51) % in sheep and 15 (11–20) % in pigs. Atelectasis correlated closely with lnPaO_2_ (R^2^ = 0.78) and shunt (R^2^ = 0.79) in sheep (P-values<0.0001). The correlation of atelectasis with lnPaO_2_ (R^2^ = 0.63) and shunt (R^2^ = 0.34) was weaker in pigs, but R^2^ increased to 0.71 for lnPaO_2_ and 0.72 for shunt 12 hours after induction of ARDS. In both, sheep and pigs, changes in atelectasis correlated strongly with corresponding changes in lnPaO_2_ and shunt.

**Discussion and Conclusion:**

In lung-healthy sheep, atelectasis correlates closely with lnPaO_2_ and shunt, when blood gases are measured during ventilation with pure oxygen. In lung-healthy pigs, these correlations were significantly weaker, likely because pigs have stronger hypoxic pulmonary vasoconstriction (HPV) than sheep and humans. Nevertheless, correlations improved also in pigs after blunting of HPV during ARDS. In humans, the observed relationships may aid in assessing anaesthesia-related atelectasis.

## Introduction

Recently, contradictory results on the effects of lung protective mechanical ventilation with low tidal volumes, positive end-expiratory pressure (PEEP) and recruitment manoeuvres during anaesthesia ventilation on the postoperative outcome have been published [1,2]. Development of atelectasis during general anaesthesia can provoke postoperative complications, but there is currently no instrument to estimate the individual amount of atelectasis in the clinical routine. Such an instrument would be the pre-requisite for individualized application of lung protective ventilator settings during anaesthesia and for monitoring of its effects. Although both, arterial partial pressure of oxygen (PaO_2_) and intrapulmonary shunt (shunt) have been suggested for estimating atelectasis and tailoring ventilator settings at the bedside or in the operating room, studies on the correlation between atelectasis and these parameters yielded inconsistent results [3–9]. The existence of strong relationships between the amount of atelectasis and PaO_2_ or shunt has not yet been consistently confirmed in clinical or experimental studies. Using the traditional definition of atelectasis in computed tomography (CT) images (i.e. CT numbers between -100 and +100 Hounsfield Units, HU), inconsistent results on the correlation between atelectasis and PaO_2_ or shunt have been reported [4,8,10–16]. This may be explained by interspecies differences in hypoxic pulmonary vasoconstriction (HPV), varying presence and activity of conditions blunting HPV (e.g. breathing of pure oxygen or inflammation) or different aetiologies of loss of lung aeration (e.g. atelectasis versus inflammatory infiltration). In vertebrates used as laboratory animals and in humans, the intensity of HPV differs significantly, as does the effect of potential inactivators on HPV [17,18]. Humans and sheep seem to be among the vertebrates in whom exposure to short-term high fractions of inspiratory oxygen (FIO_2_) blunts HPV [8,18–22], while pigs seem to have a more intense HPV, which is not that easily responding to inactivators [19,21]. If these differences would actually apply, there should be a strong correlation between atelectasis and oxygenation or shunt, respectively, in humans or sheep breathing pure oxygen, while this correlation should be weaker in pigs, as long as other conditions blunting HPV such as intense inflammation are absent. As systemic inflammation is usually absent in elective surgical cases and the FIO_2_ may be increased to 1.0 for short-periods in the vast majority of patients during anaesthesia ventilation, a reliable correlation between PaO_2_ or shunt, respectively, and atelectasis, could help to estimate relevant amounts of the latter.

We hypothesized that, by combining whole-lung CT assessment of atelectasis and short-term ventilation with pure oxygen in lung-healthy animals, we could demonstrate strong correlations between atelectasis and both, oxygenation and shunt for sheep (behaving similar to humans) and weaker correlations, if any, for pigs. The confirmation of these hypotheses would lend support to the use of these relationships for estimating atelectasis and for individualized application of lung protective ventilation in lung-healthy patients undergoing general anaesthesia.

## Methods

### Ethics statement

The governmental animal ethics and welfare committee approved the study (Landesdirektion Sachsen, Dienststelle Leipzig, Leipzig, Germany; reference numbers TVV22/04 (sheep) and TVV35/11 (pigs)). The handling of animals was in accordance with the NIH principles of laboratory animal care, all efforts were made to minimize suffering and the ARRIVE guidelines [23] were followed (see S1 ARRIVE Checklist). At the end of all experiments, animals were killed by intravenous injection of 2 g thiopental and 50 ml potassium chloride (1 M).

In order to reduce the number of animals involved in experiments, we did not perform dedicated experiments in pigs but used suitable data of another study of our group on the effects of different lung-protective ventilation strategies in experimental acute respiratory distress syndrome (ARDS). From this latter study in 23 pigs, baseline measurements (anaesthesia-related atelectasis in otherwise uninjured lungs, see below) and measurements after induction of experimental ARDS were used for the present study.

### Experiments in sheep

Eleven sheep (mean weight 68, standard deviation (SD) 8 kg) were anaesthetized using intravenous infusions of propofol (2–5 mg × kg^-1^ × h^-1^) and sufentanyl (boluses of 0.6 μg × kg^-1^) and paralyzed (bolus of 8 mg pancuronium). Tracheostomy was performed. Development of atelectasis was facilitated by commencing mechanical ventilation with pure oxygen, low tidal volumes (4–6 ml × kg^-1^ body weight) and without positive end-expiratory pressure (PEEP) (Oxylog 2000, Draeger, Lübeck, Germany). Arterial, central venous and pulmonary artery catheters were introduced using sterile techniques, the correct position of the latter was confirmed by CT scans. Fluid replacement not exceeding one litre of Ringer's lactate over the whole experiment and boluses of an anti-hypotensive drug (cafedrine/theodrenaline [24], Akrinor, AWD.pharma, Radebeul, Germany) were administered to maintain the mean arterial blood pressure above 65 mmHg. No other protocol-driven hemodynamic interventions such as continuous infusion of vasopressors were used.

After instrumentation and transportation to the CT-suite, the mechanical ventilator was changed (Servo 900D, Siemens-Elena, Solna, Sweden) without changing ventilator settings, arterial and mixed-venous blood gas were measured during ventilation with pure oxygen, and CT-scanning was performed during end-expiratory hold. Until CT, the "atelectasis-promoting" type of mechanical ventilation had been applied for approximately 60 minutes during instrumentation and transportation.

In four of the sheep, we performed additional measurements (CT scan and blood gases) after a recruitment manoeuvre (RM) and subsequent application of PEEP of 10 cmH_2_O. If atelectasis still persisted on CT (three sheep), measurements were repeated after applying another RM and PEEP of 20 cmH_2_O to achieve full lung recruitment. The RM consisted of pressure-controlled ventilation with 40 cmH_2_O PEEP and 60 cmH_2_O peak inspiratory pressure for two minutes [25]. After changes in airway pressure, we waited for 10 minutes to allow for stabilization before obtaining measurements.

A flowchart of the study protocol is provided as a supplement (see S1 Protocol).

### Experiments in pigs

Twenty-three pigs (mean weight 37, SD 5 kg) were studied in an experimental operating room equipped with a CT-scanner. Intravenous infusions of midazolam (1–5 mg × kg^-1^ × h^-1^), propofol (2–5 mg × kg^-1^ × h^-1^), ketamine (10–20 mg × kg^-1^ × h^-1^), and fentanyl (5–8 μg × kg^-1^ × h^-1^) were used for providing anaesthesia. Tracheostomy was performed, 0.15 mg × kg^-1^ × h^-1^ pancuronium was given for muscle relaxation, and mechanical ventilation was commenced with low tidal volumes (6 ml × kg^-1^ body weight) and low PEEP (5 cmH_2_O) (EVITA XL, Draeger, Lübeck, Germany). Arterial and pulmonary artery catheters were introduced using sterile techniques, the correct position of the latter was confirmed during CT. Fluid replacement not exceeding 1.5 litres over the first 12 hours of the experiment and boluses of an anti-hypotensive drug (cafedrine/theodrenaline [24], Akrinor, AWD.pharma, Radebeul, Germany) were administered to maintain the mean arterial blood pressure above 65 mmHg. No other protocol-driven hemodynamic interventions such as continuous infusion of vasopressors were used. After instrumentation was completed, the individual maintenance FIO_2_ was adjusted between 0.3 and 0.5 to achieve a peripheral oxygen saturation above 90% and baseline blood-gas measurements (arterial and mixed-venous) performed after 5 minutes of equilibration at the maintenance FIO_2_. The FIO_2_ was then increased to 1.0 and, after 5 minutes, another set of blood-gas measurements (arterial and mixed-venous) was taken and a baseline CT-scan performed. This baseline CT showed atelectasis in all pigs included in this study. Because these pigs subsequently underwent induction of experimental ARDS by repeated tracheal instillation of hydrochloric acid (until PaO_2_ remained below 200 mmHg with FIO_2_ of 1 for 30 minutes) and prolonged subsequent mechanical ventilation with PEEP levels (between 5 and 26 cmH_2_O) for 24 hours, another data set was available which was pertinent to the present study. We could only use 19 of the initial 23 pigs, because four pigs had to be excluded: one developed a pneumothorax after induction of ARDS, one died of hyperkalaemia and renal failure and data for two pigs is unavailable due to CT malfunction. After induction of ARDS, lung protective ventilation with low tidal volumes according to the ARDS network lower PEEP protocol and two different open-lung approaches were compared. The ARDS network protocol resulted in low PEEP values, while the open-lung protocols used individually titrated high PEEP using either oxygenation or electrical impedance tomography as the surrogate for "optimal" PEEP [25–27]. These different ways of individualized PEEP selection resulted in a group of pigs with widely varying magnitudes of non-aerated lung. Except for the way PEEP was individualized, the supportive treatment of the pigs in the three groups did not differ. Because we used data from this experiment from a single measurement point after 12 hours of ventilation for ARDS only, and because we do not perform any between-group comparison of data, no reference is made to further details of the experiment. The 12 hours measurement point was chosen, because it was characterized by the presence of an intense inflammatory response, which developed after induction of ARDS during lung protective ventilation. The data obtained in this situation include blood-gas measurements at maintenance FIO_2_ and FIO_2_ = 1, as well as CT-data.

A flowchart of the study protocol is provided as a supplement (see S1 Protocol).

### Blood-gas measurement and calculation of shunt

Mixed-venous and arterial blood samples were analysed immediately (ABL 800, Radiometer Copenhagen, Denmark). Berggren’s method was used to calculate shunt [28].

### CT analysis

Two multislice CT scanners were used, a Somatom Volume Zoom (120 kV tube voltage, 165 mA tube current, 4 × 2.5 mm collimation; Siemens, Erlangen, Germany) for the sheep experiment and a Philips MX8000 IDT 16 (120 kV tube voltage, 170 mA tube current, 16 × 1.5 mm collimation; Philips Medical Systems, Hamburg, Germany) for the experiments in pigs. Contiguous images were reconstructed with either 10 mm slice thickness and the standard filter “B40f” (Siemens scanner) or 6 mm thickness and the standard filter “B” (Philips scanner). No contrast medium was used.

The Osiris software (University Hospital Geneva, Switzerland) was used for manual segmentation of the lung parenchyma in CT images. Appropriate window levels and widths for lung parenchyma (-500/1,500 HU) or mediastinum (50/250 HU) were used for displaying CT-images during segmentation. Major hilar vessels and bronchi were manually excluded. In aerated lung regions, a cut-off value of -350 HU was used to aid the exclusion of partial volume effects [25,29–32]. Atelectatic lung regions were segmented manually using anatomical knowledge.

For the entire lung, the total lung volume (V_total_), mass (M_total_), and the masses and volumes of differently aerated lung compartments were calculated voxel-by-voxel as previously described [3,4,33–35]. M_total_ and V_total_ were calculated from all voxels within the -1,000 to +100 HU range. The following HU-ranges were used to obtain the masses of differently aerated lung compartments, which were calculated as percentage of M_total_: nonaerated (atelectasis, -100 to +100 HU [36]), poorly aerated (-101 to -500 HU); normally aerated (-501 to -900 HU) and hyperaerated (-901 to -1,000 HU) [35]. To re-evaluate the results of previous studies, we also quantified atelectasis by expressing the volume of voxels corresponding to atelectatic lung as percentage of V_total_. Moreover, a previously described, alternative definition of atelectasis was tested by extending the HU-window to -200 to +100 HU (instead of -100 to +100 HU) [8,36]. For the data set of pigs with ARDS, where non-aerated lung tissue consists of atelectasis, oedema and consolidation, the term “atelectasis” was kept in order to avoid unnecessary complexity.

### Statistical analysis

Results are given as median and interquartile range (IQR), unless otherwise stated. We logarithmically transformed PaO_2_ values (lnPaO_2_) to linearize its relationship with shunt, which also ensures fulfilment of basic assumption of linear regression [3,8,14]. Correlations were analysed using linear regression. Bland-Altman plots were used to assess the bias and limits of agreement (LOA) for comparing the fraction of atelectatic lung (causing shunt) and the actual intrapulmonary shunt fraction, calculated based on blood gases. This application of difference plots is an extension of the method described by Bland and Altman and has been used for similar analyses by others [14,37,38]. Bias values were compared by Mann-Whitney tests. We tested for difference in slopes of regression lines for the same parameters in pigs and sheep using analysis of covariance (ANCOVA). We calculated and analysed differences between to measurements in the same pigs (after induction of atelectasis and 12h after induction of ARDS). Statistical analysis of within- and between subject correlations of repeated measurements after recruitment manoeuvres in sheep were omitted due to the low number of animals (n = 4 for PEEP 10, n = 3 for PEEP 20) involved. Statistical analyses were performed using SPSS 20 (SPSS, Munich, Germany) and Graph-Pad Prism 5 (GraphPad Software, La Jolla, CA). Statistical significance was assumed if P < 0.05.

## Results

CT analysis revealed that in all sheep and pigs, atelectasis had developed in otherwise normal lungs during "atelectasis-promoting" mechanical ventilation. During clinical examination prior to the experiments, no animal showed signs or symptoms of infection or inflammation. There were no radiological or clinical signs of pneumonia. The median M_total_ of the atelectatic but otherwise uninjured lungs was 768 (715–884) g in sheep and 543 (503–583) g in pigs.

Further gas exchange and quantitative CT results are given in Table 1.

**Table 1:** Results from quantitative computer tomography and blood gas analysis. Values are given as median (interquartile range). Data for sheep were obtained for atelectatic (first column) and recruited lungs. Data in the second column were obtained after applying a recruitment manoeuvre (RM) and subsequent ventilation with PEEP of 10 cm H_2_O for 10 minutes. Data in the third column were obtained after applying another RM and ventilation with PEEP of 20 cm H_2_O for 10 minutes. Pigs were studied during baseline conditions (atelectasis in otherwise normal lungs) and 12 hours after induction of acute respiratory distress syndrome (ARDS). The PEEP in the ARDS column was chosen according to different lung protective ventilation strategies. N, number of animals studied; V_total_, total lung volume; M_total_, total lung mass; M_hyper_, mass of the hyperaerated (-901 to -1000 HU); M_normal_, mass of the normally aerated (-501 to -900 HU); M_poor_, mass of the poorly aerated (-101 to -500 HU); M_atelectasis_, mass of the atelectatic lung compartment (-100 to +100 HU). Weights of differently aerated lung compartments were calculated as percentage of M_total_. Atelectasis was also calculated as volume and expressed as percentage of V_total_. All blood gases were obtained after short-term ventilation with pure oxygen for five minutes. We transformed PaO_2_ values logarithmically (lnPaO_2_) to linearize the relationship between PaO_2_ and atelectasis. Shunt was calculated using Berggren’s approach. As the effects of PEEP or RM on lung aeration were no endpoints of the present study and subgroups were very small, statistical between-group comparison was omitted.

	*Sheep*			*Pigs*	
Lung condition	atelectasis	recruited	recruited	atelectasis	ARDS
n =	11	4	3	23	19
PEEP (cmH_2_O)	0	10	20	5	18 (8–21)
V_total_ (ml)	1469 (1393–1576)	2903 (2190–3516)	3397 (3237–3927)	1116 (1022–1228)	2004 (1339–2431)
M_total_ (g)	768 (715–884)	863 (825–876)	855 (782–862)	543 (503–583)	862 (799–913)
M_atelectasis_ (%)	26 (16–47)	5 (3–8)	0 (0–1)	18 (13–23)	18 (14–61)
M_poor_ (%)	37 (28–47)	20 (15–32)	6 (6–8)	41 (39–46)	31 (21–35)
M_normal_ (%)	36 (30–42)	67 (46–77)	86 (82–86)	38 (36–43)	46 (20–53)
M_hyper_ (%)	0 (0–0)	0 (0–1)	2 (0–2)	0 (0–0)	0 (0–0)
V_atelectasis_(%)	12 (6–26)	2 (1–3)	0 (0–0)	9 (7–11)	8 (6–38)
PaO_2_ (mmHg)	242 (106–414)	537 (457–550)	572 (556–584)	480 (437–514)	455 (293–506)
lnPaO_2_	5.4 (4.7–6.0)	6.3 (6.1–6.3)	6.4 (6.3–6.4)	6.2 (6.1–6.2)	6.1 (5.7–6.2)
PaCO_2_ (mmHg)	58 (51–65)	44 (42–48)	44 (43–48)	50 (45–56)	61 (53–65)
Shunt (%)	39 (29–51)	19 (14–24)	11 (8–14)	15 (11–20)	11 (7–18)

### Correlation of shunt, oxygenation and atelectasis in sheep and pigs

**Fig 1:**
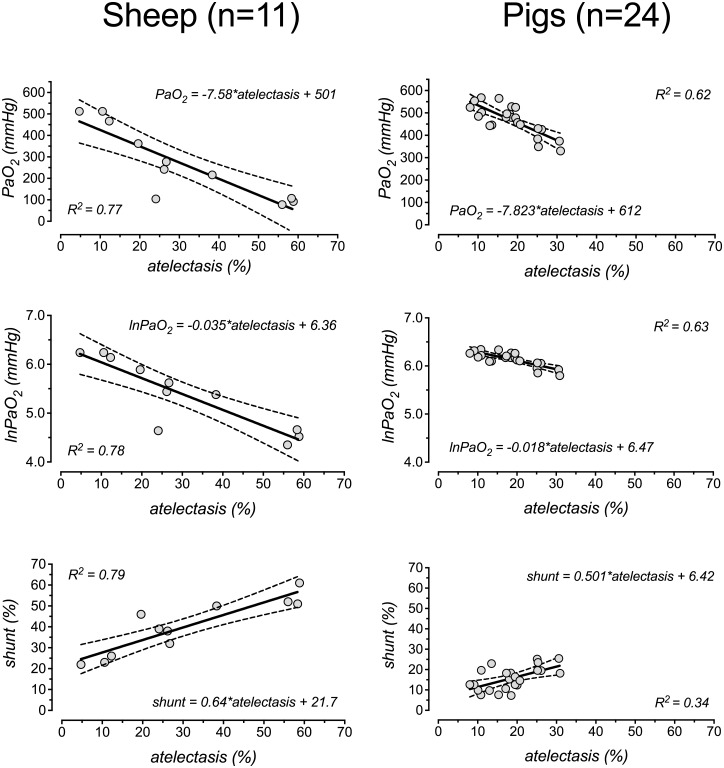
Correlation between atelectasis, oxygenation and shunt. Linear regression of raw PaO_2_ (upper row), ln-transformed PaO_2_ (lnPaO_2_, second row) and intrapulmonary (Bergren’s) shunt (lower row), respectively, on the amount of atelectasis (percentage of total lung mass). Only data points from the “atelectasis” columns in Table 1 were used. Berggren’s shunt was calculated according to [28]. We transformed PaO_2_ values logarithmically (lnPaO_2_) to linearize the relationship between PaO_2_ and atelectasis.

In sheep, regression analyses of shunt, untransformed PaO_2_ and lnPaO_2_ on atelectasis (using only the data points of the “atelectasis” columns in Table 1) showed strong correlations between these parameters (R^2^ values for correlation with atelectasis were 0.77 for PaO_2_, 0.78 for lnPaO_2_ and 0.79 for shunt; all P values < 0.0001, Fig 1).

In pigs, the correlations between atelectasis and PaO_2_ (R^2^ = 0.62, P<0.0001), between atelectasis and lnPaO_2_ (R_2_ = 0.63, P<0.0001) and between atelectasis and shunt (R^2^ = 0.34, P = 0.0034) were considerably weaker (Fig 1).

The regression equations are given in Fig 1. The slopes of the regression lines for regression of PaO_2_ and lnPaO_2_ on atelectasis were significantly less steep for pigs than for sheep (P<0.0001). Slopes for the regression of shunt on atelectasis did not differ significantly between sheep and pigs (P = 0.6).

### Influence of recruitment manoeuvres in sheep

After RMs and increases in PEEP in a subgroup of 4 sheep, we observed that the reduction of atelectasis was clearly associated with increments in PaO_2_ and lnPaO_2_ and reductions in shunt (Table 1, statistical tests were omitted).

### Influence of ARDS and prolonged ventilation in pigs

**Fig 2:**
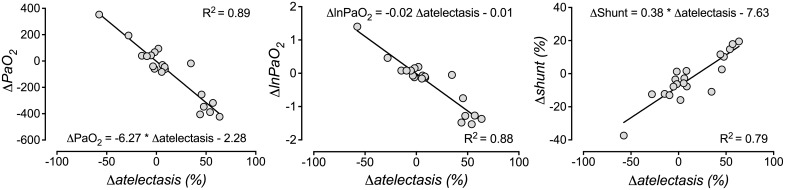
Correlation between changes of oxygenation, shunt and atelectasis in pigs. The differences (deltas) between the two measurement points in pigs (atelectasis in otherwise normal lungs and 12h after induction of ARDS) for PaO_2_, lnPaO_2_, shunt and atelectasis were calculated. These repeated measurements were available only for pigs (N = 19). Linear regression of delta-PaO_2_ (ΔPaO_2_, left panel), delta-lnPaO_2_ (ΔlnPaO_2_, central panel) or delta-shunt (Δshunt, right panel) on the changes in atelectasis (Δatelectasis) was performed. Blood gases were obtained after short-term ventilation with pure oxygen for five minutes. In this figure, atelectasis refers to real atelectasis as well as to the non-aerated lung tissue after induction of ARDS and was quantified as percentage of Mtotal (-100 to 100 HU in computer tomography). Intrapulmonary (Berggren’s) shunt was calculated according to [28]. We transformed PaO_2_ values logarithmically (lnPaO_2_) to linearize the relationship between PaO_2_ and atelectasis.

Lung injury by instillation of hydrochloric acid led to impairment of oxygenation and lung mechanics compatible with the current criteria for severe human ARDS [39] in all animals (PaO2 at FIO2 = 1.0 was 81 (66–97) mmHg at the diagnosis of ARDS). Measurements in pigs 12 hours after induction of ARDS showed an increased correlation between PaO_2_ and atelectasis (R^2^ = 0.79, P<0.0001), lnPaO_2_ and atelectasis (R^2^ = 0.72, P<0.0001) and between atelectasis and shunt (R^2^ = 0.75, P<0.0001) (see also Table 1). Also in pigs, the differences (delta) between the two measurement points in pigs (atelectasis in otherwise normal lungs and 12h after induction of ARDS) for PaO_2_, lnPaO_2_ or shunt correlated well with the changes in atelectasis between both measurement points (R^2^ = 0.89, 0.88 and 0.79 for the regression of PaO_2_, lnPaO_2_ or shunt, respectively, on atelectasis; P<0.0001; Fig 2).

### Agreement of shunt and atelectasis

**Fig 3:**
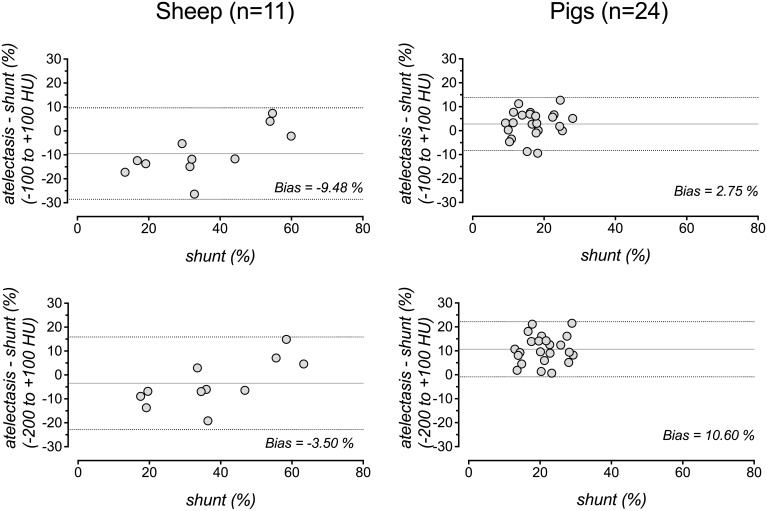
Agreement of shunt and atelectasis. Bland-Altman plots for analysis of the agreement of intrapulmonary (Berggren’s) shunt and atelectasis, when the latter was quantified by analysis of whole-lung CT and expressed as percentage of the total lung mass for sheep (left) and pigs (right) using an atelectasis definition of -100 to +100 HU (top) or an extended range of -200 to +100 HU (bottom). Shunt is plotted on the x-axis because it is considered the gold standard. The difference plotted on the y-axis was calculated by subtraction of shunt from atelectasis. Solid line: mean difference (bias), dashed lines: 95% limits of agreement (mean difference ± 1.96 SD). Blood gases were obtained after short-term ventilation with pure oxygen for five minutes.

Except for large amounts of atelectasis (>50%), shunt systematically exceeded atelectasis in sheep but not in pigs. The bias between shunt and atelectasis was -9.5% (LOA -28.6 to 9.6%) and 2.8% (LOA -8.3 to 13.8%) in sheep and pigs, respectively (Fig 3).

### Influence of parameters of quantitative CT analyses

If, instead of the percentage of M_total_, atelectasis was calculated as the percentage of V_total_, the R^2^ values decreased to 0.62, 0.67, and 0.7 for the regression of PaO_2_, lnPaO_2_ and shunt, respectively, on atelectasis in sheep and to 0.56, 0.59 and 0.18 for the regression of PaO_2_, lnPaO_2_, and shunt, respectively, on atelectasis in pigs. The Bland-Altman bias characterizing the numerical agreement of shunt and atelectasis suggested considerable underestimation of atelectasis, when it was expressed as percentage of V_total_; the bias was -22.7% (LOA -38.4 to -7.0) for sheep and -5.8% (LOA -16.1 to 4.4) for pigs. The bias values for the agreement between shunt and atelectasis defined as percentage of V_total_ differed significantly from those for the agreement between shunt and atelectasis defined as percentage of M_total_ (both P values ≤0.001).

If the HU-window defining atelectasis was extended to -200 to +100 HU (instead of -100 to +100 HU), the R^2^ value for the regression of lnPaO_2_ on atelectasis decreased from 0.63 to 0.54 for pigs but was similar (0.78 to 0.8) for sheep. The bias (LOA) between shunt and atelectasis was -3.5% (-22.9 to 15.8%) and 10.6% (-0.9 to 22.1%) in sheep and pigs, respectively, for this extended HU-window.

### Effects of inspired oxygen concentration in pigs

All correlations presented so far were based on blood gases obtained after short-term ventilation with pure oxygen for five minutes. In contrast, when pigs were ventilated with maintenance FIO_2_ of 0.3 to 0.5, R^2^ values for correlation with atelectasis were worse than those observed for pure oxygen, namely 0.43 for PaO_2_, 0.5 for lnPaO_2_ and 0.55 for shunt in uninjured lungs with anaesthesia-related atelectasis. A similar effect was observed for measurements at maintenance FIO_2_ (0.3 to 0.5) 12h after induction of ARDS: the respective R^2^ were 0.47 for PaO_2_, 0.47 for lnPaO_2_ and 0.28 for shunt.

## Discussion

We found a strong correlation between atelectasis and both oxygenation and shunt in sheep with clinically relevant amounts of anaesthesia-related atelectasis in otherwise normal lungs during mechanical ventilation with pure oxygen. In a subgroup of sheep, changes in atelectasis after lung recruitment and application of PEEP were strongly correlated to changes in both oxygenation and shunt. The respective correlations were considerably weaker in lung-healthy pigs with anaesthesia-related atelectasis, which could be attributed to a stronger hypoxic pulmonary vasoconstriction. Importantly, different measures supposed to blunt hypoxic pulmonary vasoconstriction (i.e. pure oxygen ventilation, induction of ARDS), resulted in improved correlations also in pigs. Our present findings extend previous findings of our group in ARDS patients to anaesthesia-related atelectasis.

Mechanical ventilation in the presence of atelectasis may be associated with an increased risk of ventilator associated complications [40–42]. One recent randomized study showed a benefit of lung protective ventilation aiming at the reduction of atelectasis and tidal recruitment on postoperative outcome in a lung-healthy population. In particular, this study showed a reduced necessity of non-invasive ventilation, reduced rate of re-intubation, and a shorter length of hospital stay compared to a conventional ventilation strategy [1]. However, these positive effects of a strategy for anaesthesia ventilation, which is aimed at reduction of atelectasis and at least partial restoration of normal end-expiratory lung volumes, could not be confirmed in another randomized trial [2]. To interpret such contradictory findings and to properly and individually indicate measures to restore lung volumes during anaesthesia ventilation, information about the individual amount of atelectasis would be helpful. Because information from imaging studies is usually not available during anaesthesia ventilation, previous studies suggested that inference about the amount of atelectasis could be made from shunt calculation or, if mixed venous blood samples are not available, from PaO_2_ measurement [3,8,10,12,14–16]. A broader implementation of such blood-gas derived assessment of atelectasis into the perioperative ventilation management, however, was hampered, among other reasons, by conflicting reports about the strength of the correlation between atelectasis and oxygenation or shunt [4,8,10–16,43].

For atelectatic lungs of sheep, whose HPV is—similar to humans—rather weak and easily abolished, our present whole-lung CT analysis confirmed the strong correlation between the amount of atelectasis and lnPaO_2_ and between the amount of atelectasis and shunt, respectively, and supports the feasibility of lnPaO_2_ for assessing atelectasis [10,12,16,19,22].

It is important, however, to notice that we found these strong correlations in healthy lungs only in sheep and that we used an optimized (although not at all new) methodology. Both, the effects of methodological as well as inter-species variations may help to explain previous contradictory results on the correlation between atelectasis and PaO_2_ or shunt. We combined the use of pure oxygen, the calculation of atelectasis as percentage of M_total_ instead of as percentage of V_total_ and, finally, the logarithmic transformation of PaO_2_. Every one of these measures had an influence on the correlations studied: First, breathing of pure oxygen eliminates venous admixture due to ventilation-perfusion mismatch and inactivates HPV [22]. If ventilation with pure oxygen was omitted and a FIO_2_ of 0.3 to 0.5 used, correlation was much weaker (only studied in pigs). Second, the expression of atelectasis as percentage of M_total_ better reflects the the true fraction of atelectatic lung tissue and thus makes comparison to the fraction of intrapulmonary shunt flow more reasonable [3,8,42]. The latter was confirmed by our Bland-Altman analyses showing significantly worse agreement between shunt and atelectasis expressed as percentage of V_total_ than between shunt and atelectasis expressed as percentage of M_total_ in sheep and pigs. Compatibly, the correlation was weaker between lnPaO_2_ and atelectasis expressed as percentage of V_total_ than between lnPaO_2_ and atelectasis expressed as percentage of M_total_. As shown in other studies, the use of lnPaO_2_ instead of PaO_2_ decreases the deviation of predicted values from the regression line especially in the middle (curved) portion of the nonlinear PaO_2_-atelectasis relationship, and better meets the basic assumptions of linear regression analysis [3,8,14]. The limited number of atelectasis and PaO_2_ measurements in the middle “curved” range of the nonlinear PaO_2_-atelectasis relationship may explain why the “raw” PaO_2_ and the lnPaO_2_ performed almost identically in our data.

Supporting inter-species differences, the correlation of lnPaO_2_ and atelectasis in healthy lungs was weaker in pigs than in sheep and the correlation between shunt and atelectasis was much less reliable in pigs (Fig 1). There are different possible explanations for these results. Firstly, the pigs showed a smaller median amount of atelectasis and the range of data points was considerably smaller. Moreover, pigs have a stronger HPV reducing the effects of atelectasis on shunt and arterial oxygenation, which is supported by the significantly different slopes of the regression lines for PaO_2_ and lnPaO_2_ versus atelectasis in pigs compared to sheep (see Fig 1 for regression equations). Both effects created a “cloud” of data points that resulted in impaired least square fitting results (Fig 1). An active HPV in lung-healthy pigs is also supported by the fact, that the fraction of shunt flow was smaller than the fraction of the lung parenchyma, which was atelectatic (Fig 3).

Interestingly, twelve hours after inducing experimental ARDS by hydrochloric acid aspiration in the same pigs, the R^2^ for the correlation between lnPaO_2_ and atelectasis rose to 0.71. This indicates an impairment of HPV during ARDS and systemic inflammation also for pigs in this study, which is in line with recent data for human ARDS, where (among others) our group showed a good correlation between non-aerated lung tissue and paO_2_ (R^2^ = 0.74) or lnPaO_2_ (R^2^ = 0.82) [8]. Interestingly, changes between the initial amount of atelectasis in healthy lungs and the amount of non-aerated lung during ARDS was strongly correlated to changes in lnPaO_2_ and shunt, respectively, also in pigs (Fig 2). This again points at the fact that, whenever HPV is blunted, changes in lnPaO_2_ or shunt may be a reasonable surrogate for changes in atelectasis (or non-aerated lung, as in ARDS).

Others suggested that the activity of HPV can be assessed by the ratio between atelectasis and shunt [4,44]. These calculations, however, are sensitive to the assumption that the amount of shunting lung units is correctly estimated by CT. As the calculation of the ratio between atelectasis and shunt is analogous to forcing the regression line for shunt versus atelectasis to go through the origin of the coordinate system, the activity of HPV may seem to depend on the amount of atelectasis as suggested by Cressoni and colleagues [4]. By quantitative analysis of whole-lung CT, however, we found that the shunt fraction (Berggren) systematically exceeded the atelectatic fraction of the lung parenchyma in sheep (bias -9.48%, Fig 3). Besides a blunted HPV, this most likely represents a true y-axis-intercept, which may be explained by true anatomical shunt (Thebesian and deep bronchial veins). Theoretically, there may also be an underestimation of the of the truly shunting lung tissue fraction when it is assessed by CT using the -100 to +100 HU window [14]. However, the use of the extended HU-window of -200 to +100 HU for definition of atelectasis in CT did not consistently improve the strength of correlation with PaO_2_ or lnPaO_2._ This confirms a previous report for homogeneous, anaesthesia-related absorption atelectasis [36], while inhomogeneously distributed, oedematous non-aerated lung tissue of ARDS patients may be better covered by the -200 to +100 HU definition [8].

Obviously, validation of our results in human patients appears necessary. However, we could not yet identify a population of lung-healthy patients who undergo CT of the lung during general anaesthesia and have an arterial catheter at the same time. Besides validation, such results would aid in providing a look-up table for approximation of the amount of atelectasis from lnPaO_2_ and/or shunt measurements during anaesthesia ventilation [8].

### Limitations of our study

Results from the atelectasis model used in our study may apply to patients with atelectasis who are ventilated during anaesthesia but may not be directly transferable to patients with oedematous and/or inflamed lungs. Moreover, sheep have reduced collateral ventilation, which may influence the potential for developing absorption atelectasis and also the matching of aeration and gas exchange [45]. Changes in the arterial partial pressure of carbon dioxide (PaCO_2_) may alter the perfusion of atelectatic lung regions. Hypocapnia, which reduces or even abolishes HPV, was avoided [46]. Further control of PaCO_2_, however, would have generated unrealistic results with limited applicability in the clinical anaesthesia setting. Although animals in the present study showed neither clinical nor radiological signs of pneumonia, an infection potentially interacting with HPV cannot be excluded. Furthermore, anaesthetic drugs may alter HPV. However, the drugs used in our study are frequently used in the clinical setting and seem to have a limited effect on HPV [47,48].

Using pure oxygen temporarily during measurement periods might be discussed controversially, since even short periods of pure oxygen ventilation can promote atelectasis and increase shunt, particularly during and after induction of anaesthesia [10,49,50]. Nevertheless, pure oxygen ventilation to blunt HPV is applied only for five minutes. Furthermore, rapid development of atelectasis as described during preoxygenation and induction of anaesthesia will be significantly reduced by application of PEEP, which is part of many contemporary concepts for anaesthesia ventilation [1,2,51]. Additionally, the gain in information about the existence and magnitude of atelectasis outweighs, at least in our opinion, the risk of some increase in atelectasis, which can be easily reversed by recruitment manoeuvres and application of PEEP [52].

The protocols of our experiments in pigs and sheep, from which the data for the current study was obtained, differ in certain points (e.g. PEEP for induction of atelectasis, type of CT scanner, additional induction of ARDS in pigs). These earlier experiments investigated different research questions and there is no overlap or duplicate publication of results.

## Conclusion

In sheep with absorption atelectasis breathing pure oxygen, we could show strong linear relationships between atelectasis and both, oxygenation and intrapulmonary shunt. After lung recruitment, reductions in atelectasis correlated with increments in oxygenation and decreases in shunt. Our results in sheep, whose HPV physiology has similarities to the human one, suggest that oxygenation and shunt could be used to estimate atelectasis. These finding extend our previous findings in ARDS patients to anaesthesia-related atelectasis. Pigs, which have a much more intense HPV than humans, did not show such strong correlations for atelectasis in healthy lungs. However, after induction of ARDS, these correlations were similarly strong for pigs and sheep.

Since arterial catheters and thus the PaO_2_ are frequently available in the perioperative setting, especially in critically ill patients or patients undergoing major surgical procedures, estimation of atelectasis based on the PaO_2_ could be an instrument to individualize lung protective ventilation and minimize atelectasis during general anaesthesia, provided that adequate measures are taken to blunt hypoxic pulmonary vasoconstriction.

## Supporting Information

S1 Protocol Flowchart of study protocol. See methods section for a detailed description of our study protocol in sheep and pigs.
(PDF)

S1 ARRIVE Checklist ARRIVE (Animal Research: Reporting In Vivo Experiments) Guidelines Checklist. See methods section and [23] for details.
(PDF)

S1 Table Raw data of pigs with atelectasis in otherwise normal lungs. Measurements obtained under ventilation with pure oxygen (values with „_1“) and at individual FIO2 (values with „_ind“).
(CSV)

S2 Table Raw data of sheep with atelectasis in otherwise normal lungs. Ventilation with pure oxygen. Measurements at baseline and after recruitment with PEEP of 10cmH2O or 20cmH2O.
(CSV)

S3 Table Raw data of pigs after induction of ARDS. Measurements obtained under ventilation with pure oxygen.
(CSV)

S4 Table Raw data of pigs 12 hours after induction of ARDS. Measurements obtained under ventilation with pure oxygen (values with „_1“) and at individual FIO2 (values with „_ind“). Pigs 23 and 14 were excluded due to malfunction of computer tomography. Pig 22 was excluded due to hyperkalaemia and renal failure. Pig 6 was excluded due to a pneumothorax. Due to mentioned reasons, measurements could only be obtained for 19 of 23 pigs studied with atelectasis in otherwise normal lungs before induction of ARDS.
(CSV)
