# Supplementary figures and images for: Correlation of Lung Collapse and Gas Exchange - A Computer Tomographic Study in Sheep and Pigs with Atelectasis in Otherwise Normal Lungs
Source: PLoS One. 2015 Aug 10;10(8):e0135272. doi: 10.1371/journal.pone.0135272 (PMC4530863; doi:10.1371/journal.pone.0135272)

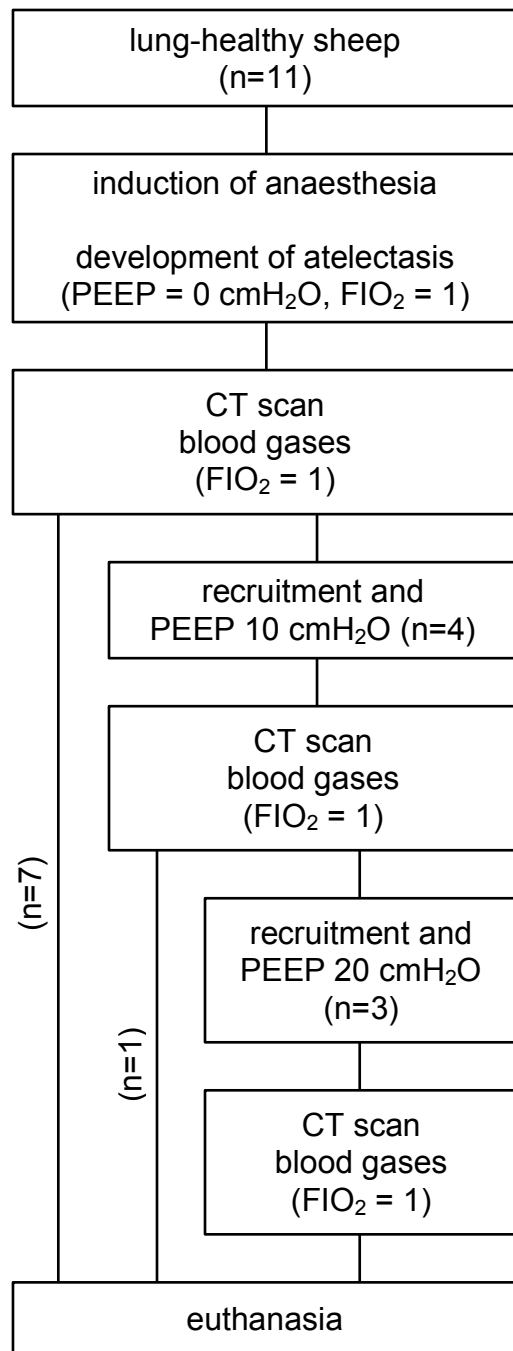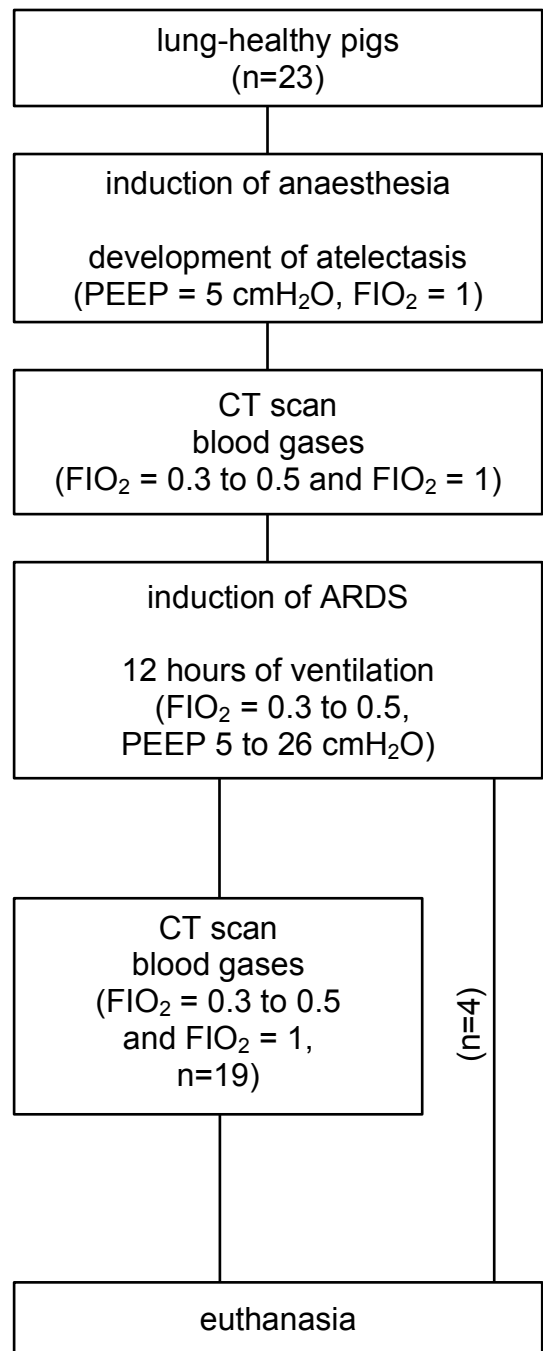

Supplement: S1 Protocol — See methods section for a detailed description of our study protocol in sheep and pigs. (PDF) [file pone.0135272.s001.pdf]
